# Chemical Composition and Antioxidant Activities of Polysaccharides from *Yingshan Cloud Mist* Tea

**DOI:** 10.1155/2019/1915967

**Published:** 2019-08-21

**Authors:** Xiang Li, Si Chen, Jing-En Li, Ning Wang, Xin Liu, Qi An, Xi-Mei Ye, Zi-Tong Zhao, Meng Zhao, Yi Han, Ke-Hui Ouyang, Wen-Jun Wang

**Affiliations:** ^1^Jiangxi Key Laboratory of Natural Product and Functional Food, College of Food Science and Engineering, Jiangxi Agricultural University, Nanchang 330045, China; ^2^College of Animal Science and Technology, Jiangxi Agricultural University, Nanchang 330045, China

## Abstract

The study was designed to investigate the chemical composition and antioxidant activities of polysaccharides from *Yingshan Cloud Mist* Tea. The chemical composition of green tea polysaccharides (GTPS) was analyzed by Fourier-transform infrared (FT-IR) spectroscopy, scanning electron microscope (SEM), thermogravimetric (TGA), gas chromatograph (GC), and high-performance gel-permeation chromatography (HPGPC). Then, the antioxidant activities *in vitro* of GTPS, effects of GTPS on body weight, and the antioxidant activities in chickens were studied. The results showed that GTPS were composed of rhamnose (Rha), arabinose (Ara), xylose (Xyl), mannose (Man), glucose (Glu), and galactose (Gal) in a molar ratio of 11.4 : 26.1 : 1.9 : 3.0 : 30.7 : 26.8 and the average molecular weight was 9.69 × 10^4^ Da. Furthermore, GTPS exhibited obvious capacity of scavenging DPPH radical, hydroxyl radical, and superoxide radical and enhanced the ferric-reducing power *in vitro*. Last, GTPS significantly increased the body weight of chickens, enhanced the T-AOC, SOD, and GSH-Px level, and decreased the content of MDA in chickens. The results indicated that GTPS might be a kind of natural antioxidant, which had the potential application in feed industry.

## 1. Introduction

Oxidative stress and its negative effects on animal body health have become an important topic in recent decades. The generation of reactive oxygen species (ROS) will result in the homeostatic imbalance when organisms are exposed to exogenous and endogenous factors [1]. Excessive ROS not only accelerate the aging process but also cause many human diseases, including cancer, reperfusion injury, and hepatic injury [2]. In order to reduce the oxidative damage of ROS, many synthetic antioxidants have been used widely nowadays. However, many reports suggest that synthetic antioxidants should be restricted due to the suspicions on carcinogenic effects related to health [3]. Natural polysaccharides are capable of preventing oxidative damage in human and animal bodies from scavenging free radicals, which are widely distributed in animals, plants, and microorganisms [4]. Therefore, there is growing attention to investigations focusing on the natural polysaccharides since a lot of data indicated that plant polysaccharides in general have strong antioxidant activities [5].

Green tea (*Camellia sinensis L.*) is one of the most popular beverages and popularly consumed because it has a basic crude tea leaves material, which is different from partially fermented and fermented tea, and keeps most of its bioactive components [6]. *Yingshan Cloud Mist* tea, a kind of green tea, specially produced in Yingshan County, Hubei Province, is very popular in the local people due to its health-promoting benefits [7]. Nowadays, many studies have reported that tea polysaccharides have a wide range of biological activities such as antioxidant, anticancer, hypoglycemic, antiobesity, antiatherogenic, antibacterial, and improving immune function [8–10]. In the previous researches, *in vitro* assays showed that green tea polysaccharides had obvious scavenging activities including DPPH radical, superoxide anion radical, and hydroxyl radical, suggesting the biomedical potential of polysaccharides of green tea [11]. Also, it was reported that green tea polysaccharides could enhance the antioxidant enzyme activities (GSH-Px, SOD, and CAT) and inhibit the lipid peroxidation in many animal organisms [12].

However, there are few reports about the application of tea polysaccharides to the feed industry. In this study, the green tea polysaccharides (GTPS) were extracted from *Yingshan Cloud Mist* Tea leaves by hot water and purified by the DEAE cellulose columns. The aim of our present study was to identify the structural features of GTPS and evaluate its antioxidant property to provide a theoretical basis for the application of GTPS in the feed industry.

## 2. Materials and Methods

### 2.1. Materials and Reagents

*Yingshan Cloud Mist* Tea was purchased from Yingshan County, Caotangchun Tea Co. Ltd. (Hubei Province, China). Dextrans of different molecular weight (T-1300, T-6000, T-12000, T-22000, T-50000, T-110000, T-400000, and T-800000) and the standard monosaccharides samples (rhamnose, fucose, arabinose, xylose, mannose, glucose, and galactose) used in this study were purchased from Sigma Chemical Co. (MO, USA). 1,1-diphenyl-2-picrylhydrazyl (DPPH), the ascorbic acid, and trichloroacetic acid solution were purchased from Aladdin Biotechnology Co. Ltd. (Shanghai, China). The commercial assay kits for GSH-PX, SOD, MDA, and T-AOC were purchased from Nanjing Jiancheng Bioengineering Research Institute (Nanjing, Jiangsu, China). All other chemicals and reagents were of analytical grade in this study.

### 2.2. Preparation of GTPS

The crude polysaccharides of the green tea were extracted according to the description of Xie et al. with some modifications [13]. First, the green tea leaves were ground by a high speed pulverizer into a fine powder and passed through a 60-mesh sieve [14]. The dried green tea powder was mixed with 80% ethanol for 24 h to remove the interference components in tea such as polyphenols, pigments, lipids, monosaccharides, and oligosaccharides. Then, the precipitates were extracted with boiling distilled water (1 : 20, *w*/*v*) in bath twice. Second, the water extracts were gathered and concentrated to 30% of their initial volume with a rotary evaporator under reduced pressure. After that, the remaining solution was precipitated with 95% (*v*/*v*) ethanol at 4°C overnight. The precipitates obtained were centrifuged at 3500 rpm for 15 min. The combined supernatant was mixed with sevag reagent (CHCl_3_/n − BuOH = 4 : 1, *v*/*v*) in order to remove the free protein [15]. Finally, after removing the sevag reagent, the aqueous layer was centrifuged at 3500 rpm for 15 min to remove the insoluble ingredients. The precipitates were dialyzed with an 8000-14000 Da dialysis bag (Solarbio, Beijing, China) to remove small molecular substances. Then, the rest of the solution was lyophilized under vacuum to obtain the crude tea polysaccharides.

After that, the crude tea polysaccharides were dissolved with double distilled water and centrifuged at 10000 rpm for 15 min to remove the insoluble impurities. The resulting supernatant was added into a DEAE cellulose column (100 × 7.0 cm, Sigma-Aldrich) eluted by 0.1 mol/L NaCl aqueous solution at a flow rate of 2.0 mL/min. The aqueous solution collected was concentrated in a rotary evaporation, dialyzed, freeze-dried, and defined as GTPS.

### 2.3. Chemical Component Analysis of GTPS

The total carbohydrate content of GTPS was determined by the phenol-sulfuric acid method with glucose as a standard. The protein content of GTPS was determined by the Bradford method using bovine serum albumin (BSA) as a standard.

### 2.4. Structure Analysis of GTPS

#### 2.4.1. Fourier-Transform Infrared (FT-IR) Spectroscopy

The FT-IR spectrum of GTPS was measured on a Nicolet iS5 FT-IR spectrometer (Thermo Fisher Scientific, USA) with a range of 400-4000 cm^−1^. The powder of GTPS (1 mg) was mixed with KBr powder (100 mg) and then pressed into 1 mm pellets for further infrared spectrometer analysis.

#### 2.4.2. Scanning Electron Microscope (SEM)

The sample powder was completely coated with gold powder under vacuum using a QUANTA 250 scanning electron microscope (FEI, USA). The morphology of GTPS was recorded by an acceleration voltage of 15 kV with an image enlargement multiple 200, 500, 2000, and 10000.

#### 2.4.3. Thermogravimetric (TGA)

GTPS were recorded by a TGA 4000 thermogravimetric analyzer (Sum, Massachusetts, USA). The powder of GTPS (3.072 mg) was put into a sample container. The gas was switched to nitrogen at 20.0 mL/min, and the sample was heated from 30°C to 600°C at 10°C/min.

#### 2.4.4. Monosaccharide Composition and Molecular Weight

The monosaccharide composition and molar ratios of GTPS were determined by a gas chromatograph system (GC-7890A, Agilent Technologies, USA) equipped with a Hp-5 capillary column (30 mm × 0.25 mm × 320 *μ*m) [16]. The molecular weight of GTPS was determined by high-performance gel-permeation chromatography (HPGPC) on an Agilent 1260 high-performance liquid chromatograph (HPLC, Agilent Corporation, USA) equipped with series of SHODEX KS-804 and KS-802 columns and a refractive index (RI) detector [17].

### 2.5. Antioxidant Activities of GTPS In Vitro

#### 2.5.1. DPPH Radical Scavenging Activity

The DPPH radical scavenging activity assay of GTPS was conducted according to Zhu et al.'s study with some slight modifications [18]. Briefly, GTPS solution was dissolved in double distilled water at various concentrations (0, 0.1, 0.2, 0.4, 0.8, 1.6, 2.0, and 2.5 mg/mL). One mL of sample solution was mixed with 1 mL 0.5 mM DPPH solution (prepared with ethanol) and shaken vigorously. The mixture was incubated in the darkness at room temperature for 30 min, and then, the absorbance of solution at 517 nm was measured by a UV–vis spectrophotometer. The ascorbic acid was included as a positive control, and the double distilled water was included as a negative control. The capability of GTPS to scavenge the DPPH radical was calculated according to the following equation:

(1) $$\begin{array}{c} \text{The DPPH radical scavenging activity rate }\left(\%\right)=\left[1-\frac{\left(A_{1}-A_{2}\right)}{A_{3}}\right]\times 100\%, \end{array}$$

where *A*_1_ was the absorbance of the GTPS sample, *A*_2_ was the absorbance of the sample solution using absolute ethyl alcohol instead of DPPH solution, and *A*_3_ was the absorbance of the negative control (using double distilled water instead of sample solution).

#### 2.5.2. Superoxide Anion Radical Scavenging Activity

The superoxide anion scavenging activity assay of GTPS was performed according to Chen and Huang with some minor modifications [19]. Briefly, 0.25 mL of the GTPS sample solution with different concentrations (0.1, 0.4, 0.7, 1.0, 2.0, 3.0, 4.0, and 5.0 mg/mL) was mixed with 1 mL of 50 mM Tris-HCl buffer solution (pH = 8.2) and incubated in a water bath at 25°C for 20 min. Then, 0.5 mL pyrogallic acid (7 mM) was added to the reaction mixture and incubated at 25°C for another 5 min. 0.25 mL HCl (8 mM) was added to the mixture to stop the reaction, and the absorbance of the mixture was measured at 325 nm. The ascorbic acid was used as a positive control, and the double distilled water was used as a negative control. The activity of GTPS to scavenge the superoxide anion was calculated as

(2) $$\begin{array}{c} \text{The superoxide anion scavenging activity rate }\left(\%\right)=\left[\left(\frac{A_{0}-A_{1}}{A_{0}}\right)\right]\times 100\%, \end{array}$$

where *A*_0_ was the absorbance of the negative control solution, and *A*_1_ was the absorbance of the samples.

#### 2.5.3. Hydroxyl Radical Scavenging Activity

The former reported method [20] with slight modifications was carried out to detect the hydroxyl radical scavenging activity of GTPS. Briefly, 0.5 mL GTPS sample solution with different concentrations (0.1, 0.4, 0.7, 1.0, 3.0, 4.0, 5.0, 7.0, and 9.0 mg/mL), 0.5 mL of 6 mM FeSO_4_, and 0.5 mL of 6 mM salicylic acid (prepared with alcohol) were added into test tubes. Then, the reaction was mixed with 0.5 mL 4 mM H_2_O_2_ and incubated at 25°C for 30 min. The absorbance of the mixture was measured at 510 nm. The ascorbic acid was used as a positive control, and the double distilled water was used as a negative control. The hydroxyl radical scavenging activity was calculated as

(3) $$\begin{array}{c} \text{Hydroxyl radical scavenging activity rate }\left(\%\right)=\frac{A_{0}-A_{1}}{A_{0}}\times 100\%, \end{array}$$

where *A*_0_ was the absorbance of the negative control solution, and *A*_1_ was the absorbance of the various GTPS samples.

#### 2.5.4. Ferric-Reducing Power Activity

The ferric-reducing power was detected according to the previous method of Chen et al. with some modifications [21]. Briefly, 2.0 mL GTPS sample solution with different concentrations (0.1, 0.4, 2.0, 3.0, 4.0, 5.0, 7.0, and 9.0 mg/mL), 2.0 mL potassium phosphate buffer (0.2 M, pH = 6.6), and 2.0 mL potassium ferricyanide (1.0%, *w*/*v*) were placed in test tubes and incubated at 50°C for 20 min. Then, 2.0 mL trichloroacetic acid solution (10%, *w*/*v*) was added to the reaction, followed by centrifugation at 3000 rpm/min for 10 min. After that, 2.0 mL supernatant was mixed with 0.5 mL of ferric chloride solution (1%, *w*/*v*) and 2.0 mL double distilled water, and then, the mixture solution was kept at room temperature for 10 min. The absorbance of the GTPS sample solution was measured at 700 nm. The ascorbic acid was used as a positive control.

### 2.6. Animal Design

Two hundred one-day-old Chongren Chickens were purchased from a local commercial hatchery and vaccinated with infectious bronchitis and Newcastle disease vaccines. Before the experiment, all chickens cages, instruments, and house environment were strictly disinfected and sterilized. After prefeeding for 20 days, all 200 healthy chickens were randomly allotted to four experimental dietary groups including 3 treatment groups and 1 normal control (NC) group. Each group had 5 replicates with 10 chickens each. The grouping arrangement was as follows: normal control group (basal diet) and 3 treatment groups (basal diet fully mixed with 200 mg/kg, 400 mg/kg, and 800 mg/kg GTPS, respectively). The whole experiment process continued for 56 days. All procedures and related protocols adopted in this experiment were approved by the Animal Care and Use Committee of Jiangxi Agricultural University (Nanchang, Jiangxi Province).

The chickens were supplemented with a standard starter basal diet (17.80 MJ/kg metabolic energy and 20% crude protein) in the period of prefeeding and a standard grower basal diet (18.50 MJ/kg metabolic energy and 12% crude protein) until the end of the experiment. The chickens were reared in battery wire cages. The basal diets in mash form and water were supplied *ad libitum* during the experiment duration. The environmental temperature of the house was maintained at about 32°C for the first 3 days and then reduced by 3°C each week until the temperature reached 24°C at the rest of the study. The chickens' house was provided with a 12 h light and 12 h dark lighting program throughout the experiment.

#### 2.6.1. Sample Collection

The body weight, average daily gain, average daily feed intake, and feed conversion ratio of 4 chicken groups (3 treatment groups and 1 normal control group) were recorded at 28 d and 56 d of the experiment.

At 28 and 56 d of this study, 3 chickens of each replicate (15 chickens per group, a total of 60 chickens) with similar weight were selected randomly after fasted for 12 h. After that, the blood samples (3-4 mL) were collected from chicken wing vein and then put into tubes. Samples were kept for 2 h at room temperature and then centrifuged (3500 rpm) for 15 min. Serum was preserved in the -80°C refrigerator for further biochemical analysis.

#### 2.6.2. Determination of Antioxidant Activities in Chickens

The antioxidant activities of superoxide dismutase (SOD), glutathione peroxidase (GSH-Px), total antioxidant capacity (T-AOC), and the concentration of malondialdehyde (MDA) were analyzed by commercial antioxidant assay kits according to Wu et al. with some modifications [22].

### 2.7. Statistical Analysis

All data was performed by the SPSS statistical software package (Version 20.0, SPSS Inc., Chicago) and analyzed by one-way analysis of variance (ANOVA). The results were expressed as the mean ± standard deviation (SD), and Duncan's multiple range tests were used to determine the differences between 3 treatment groups and the normal control group. The significant difference was set at the level of *P* < 0.05. The number of chickens in 4 groups (*n* = 15, 3 chickens of 5 replicates) was used as the experiment unit for data analysis.

## 3. Results

### 3.1. Structural Results of GTPS

#### 3.1.1. Chemical Compositions and FT-IR Spectroscopy

The final yield of GTPS was 2.5%. The total carbohydrate content of GTPS was 67.36%. The content of protein of GTPS was 5.67%.

**Figure 1:**
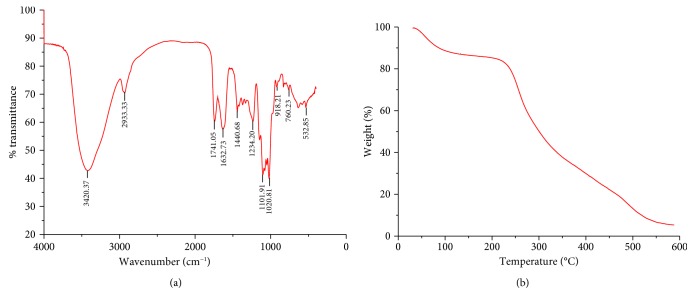
FT-IR and TGA analysis of GTPS. (a) Spectrum of GTPS purified from green tea in the range of 4000-400 cm^−1^. (b) TGA results of GTPS.

The FT-IR spectrum of GTPS was examined and presented in Figure 1(a). The spectrum results were recorded and ranged from 400 cm^−1^ to 4000 cm^−1^. A broad and intense absorption peak at around 3420 cm^−1^ was attributed to the stretching vibration of O-H groups [23]. The weak peak signal at 2933 cm^−1^ was recognized as the C-H stretching vibration [24]. The absorption at approximately 1741 cm^−1^ and 1440 cm^−1^ indicated the presence of the carboxyl groups and carbonyl groups, respectively, which proved the characteristic existence of uronic acids in GTPS [25]. The relative weak signal peak appeared at about 1632 cm^−1^ was the characteristic absorption peak of protein, and several weak peaks observed from 1400 to 1200 cm^−1^ were attributed to C-H bonds of polysaccharide. Furthermore, the strong and sharp absorption bands from 1200 cm^−1^ to 1000 cm^−1^ were assigned to C-O-C stretching vibration of glycosidic bonds and the C-O-H group. However, the weak peak at 918.21 cm^−1^ was *β*-pyranose ring peak. The bands at 532 cm^−1^ and 760 cm^−1^ were assigned to the skeletal modes of pyranose rings in the monosaccharide of GTPS.

#### 3.1.2. TGA Analysis

The results of TGA were presented in Figure 1(b). As shown in the TGA curves, GTPS had a decomposition stage. The first decomposition process started from around 35°C to 100°C, which was assigned to the associated water. The second decomposition process started from around 100°C to 200°C, which was assigned to the side groups. The third decomposition process started from around 200°C to 550°C, which was assigned to the main chains. From 550°C to 600°C, the sample weight was basically stable.

#### 3.1.3. Scanning Electron Microscope

**Figure 2:**
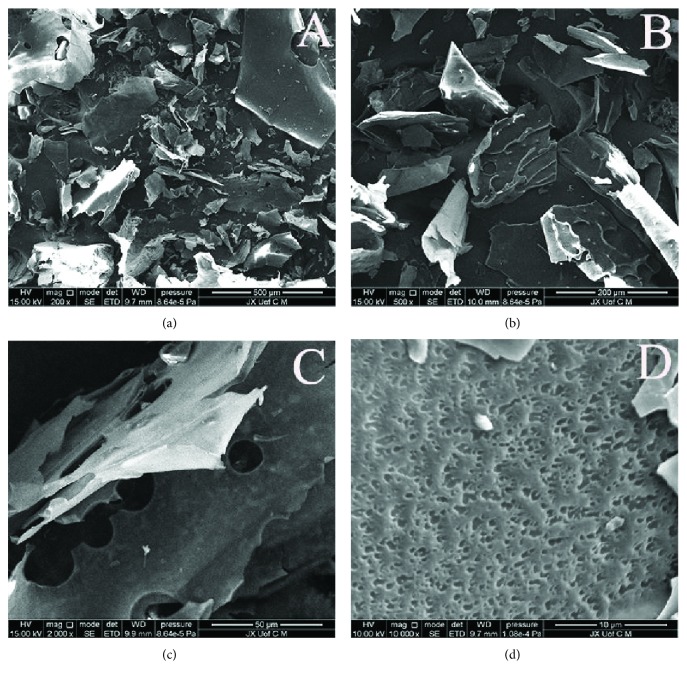
Scanning electron micrographs (SEM) of GTPS: (a) GTPS (200x), (b) GTPS (500x), (c) GTPS (2000x), and (d) GTPS (10000x).

SEM images were shown in Figure 2. It showed that the results at 200-fold and 500-fold magnification (Figure 2(a) and Figure 2(b)) indicated that the surface of GTPS was smooth and mainly exhibited sheet and leaf structure. The shapes of the sample appeared an irregular, large lamellar state, and each sheet had some stripes. Furthermore, the images at 2000-fold magnification (Figure 2(c)) revealed that there were small holes on the surfaces of polysaccharide sheets. Seen from the results at 10000-fold magnification (Figure 2(d)), GTPS exhibited a tight, rough, and uneven surface including some solid blocks.

#### 3.1.4. Monosaccharide Composition and Molecular Weight

**Figure 3:**
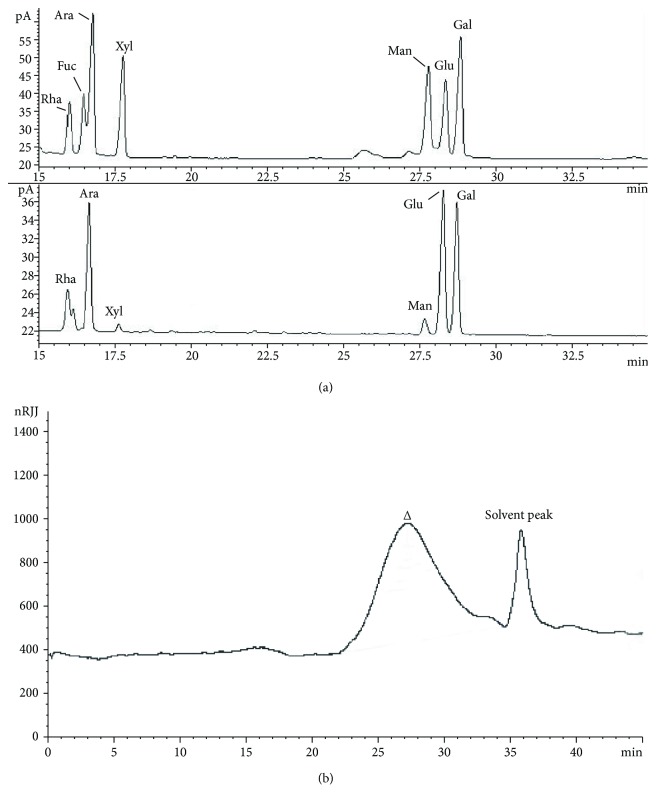
GC chromatograms (a) and HPGPC profile (b) of GTPS.

The monosaccharide compositions of GTPS were shown in Figure 3(a). According to the retention time of acetate derivative of monosaccharide standard in GC spectra, the retention time of seven standard monosaccharide samples (Rha, Ara, Xyl, Man, Glu, and Gal) was 16.093, 16.624, 16.599, 27.632, 28.17, and 28.629, respectively. GTPS was composed of rhamnose, arabinose, xylose, mannose, glucose, and galactose in a molar ratio of 11.4 : 26.1 : 1.9 : 3.0 : 30.7 : 26.8.

As shown in Table 1 and Figure 3(b), the analysis results exhibited that according to the calibration curve, the average molecular weight (Mw) of GTPS was calculated to be 9.69 × 10^4^ Da according to the calibration curve.

### 3.2. Effects of GTPS on Growth Performance in Chickens

As shown in Table 2, compared with the initial body weight of the chickens after the prefeeding period, the results showed no significant difference (*P* > 0.05) among the 4 experimental dietary groups, which indicated that the distribution was reasonable. At the 28 d and 56 d of the experiment, body weight, average daily gain, and average daily feed intake of 3 treatment groups were considerably higher than that of the normal control group (*P* < 0.05). However, the feed conversion ratio showed no significant difference (*P* > 0.05), which indicated that GTPS failed to induce any substantial effect on feed conversion ratio across the experiment. As can be seen, it demonstrated that GTPS had the potential effect in promoting weight gain in chickens with the increasing of the concentration of the polysaccharides (200 mg/kg, 400 mg/kg, and 800 mg/kg).

### 3.3. Analysis of Antioxidant Activities of GTPS In Vitro

**Figure 4:**
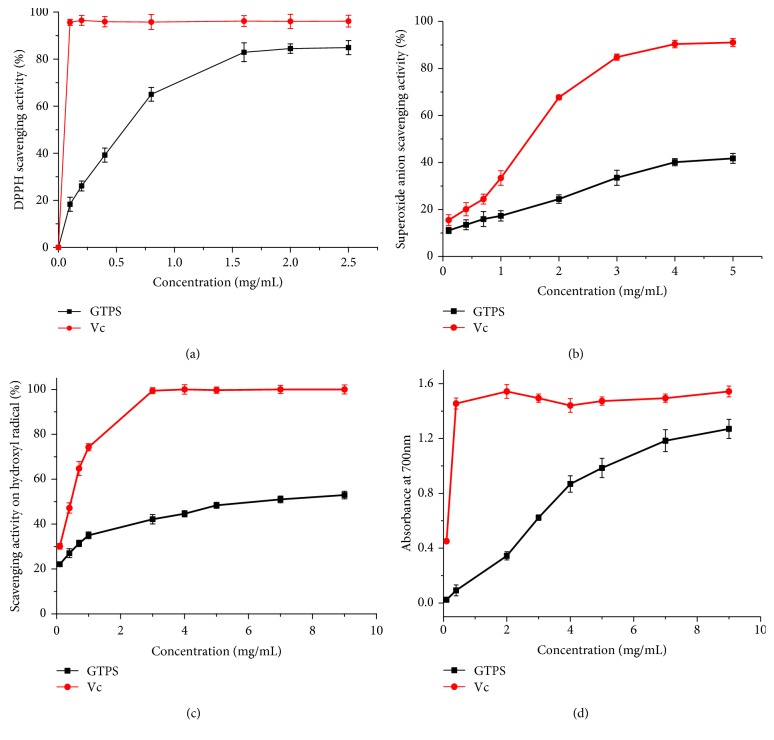
*In vitro* antioxidant activity of GTPS and vitamin C. (a) DPPH radical scavenging activity, (b) hydroxide radical scavenging activity, (c) superoxide anion radical scavenging activity, and (d) ferric-reducing power activity.

The antioxidant activities of GTPS were determined by ferric-reducing power assay and scavenging activities for 3 kinds of radicals including hydroxyl, superoxide anion, and DPPH radicals (Figure 4). As shown in Figure 4(a), GTPS exhibited dose-dependent DPPH scavenging activity. At a concentration of 2.5 mg/mL, the DPPH free radical scavenging rates of GTPS and ascorbic acid were 84.90% and 96.15%. The half maximal inhibitory concentration (IC_50_) of the scavenging capacity of GTPS and ascorbic acid was 0.6 mg/mL and 0.05 mg/mL, respectively. As illustrated in Figure 4(b), GTPS and ascorbic acid also showed superoxide radical-scavenging effects in a dose-dependent manner. At the concentration of 5.0 mg/mL, the superoxide radical scavenging activities of GTPS and ascorbic acid were 41.73% and 91.05%. The GTPS showed a relatively high capacity of eliminating superoxide anion radical, and the IC_50_ of ascorbic acid was 1.50 mg/mL. The scavenging effect on hydroxyl radical was shown in Figure 4(c). The scavenging efficiency of GTPS and ascorbic acid at 9.0 mg/mL was 52.85% and 100%, respectively. The IC_50_ of GTPS and ascorbic acid was 8.9 mg/mL and 0.45 mg/mL, respectively. As can be seen from Figure 4(d), ascorbic acid had the best reducing capacity, and the reducing capacity of GTPS had a considerable change with the increasing of concentration. In general, GTPS had excellent antioxidant activity *in vitro*.

### 3.4. Analysis of Antioxidant Activities of GTPS in Chickens

**Figure 5:**
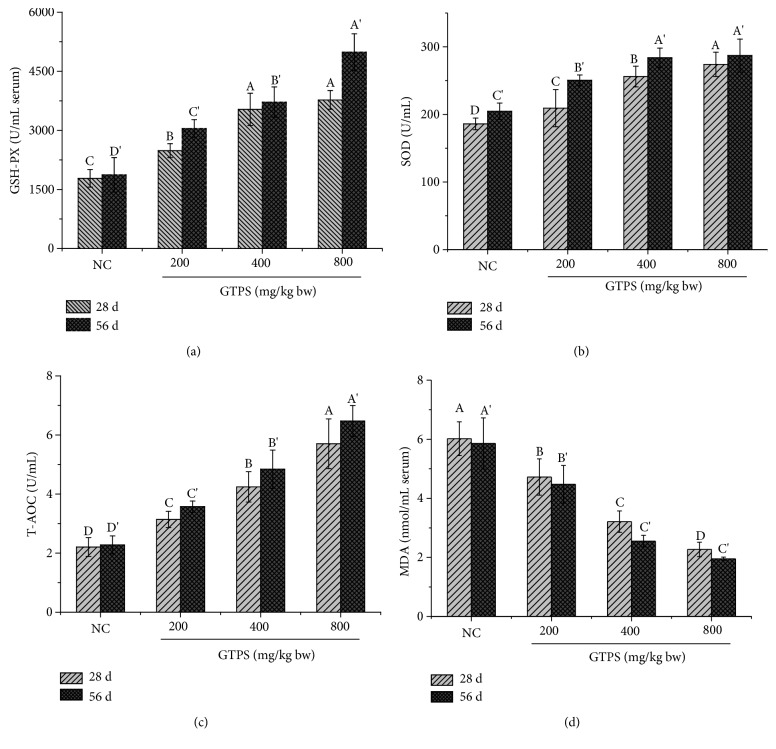
Effects of dietary GTPS on serum antioxidant activities of GSH-Px, SOD, T-AOC, and MAD levels. Broilers were divided into four groups: the high-dose group (HDG, 800 mg/kg), middle-dose group (MDG, 400 mg/kg), low-dose group (LDG, 200 mg/kg), and normal control group (NC, basal diet without GTPS). Values were means ± SD, *n* = 15. a, b, c, d (28 d) and a′, b′, c′, d′ (56 d): bars with different letters were significantly different (*P* < 0.05) in reference to Duncan's multiple tests.

In this study, as shown in Figures 5(a)–5(c), at 28 d and 56 d of the experiment, the antioxidant activities of GSH-Px, SOD, and T-AOC of 3 treatment groups supplemented with GTPS were significantly increased (*P* < 0.05) comparing with the normal control group. Meanwhile, the level of MDA (Figure 5(d)) was significantly decreased (*P* < 0.05). However, with the increasing of experiment period (28 d and 56 d) and with the increasing of the concentration of GTPS (200 mg/kg, 400 mg/kg, and 800 mg/kg), the capacities of GSH-Px, SOD, and T-AOC were further enhanced (*P* < 0.05), while the content of MDA was reduced (*P* < 0.05). At 56 d of the study (Figure 5(b)), the level of SOD for MDG and the HDG had no significant difference (*P* > 0.05), which suggested that 400 mg/kg GTPS might be the most appropriate concentration.

## 4. Discussion

In our study, GTPS was successfully extracted from *Yingshan Cloud Mist* Tea, and its chemical composition was investigated. It was found that GTPS was a kind of protein-bound polysaccharide and composed of rhamnose, arabinose, xylose, mannose, glucose, and galactose in a molar ratio of 11.4 : 26.1 : 1.9 : 3.0 : 30.7 : 26.8 and the molecular weight was 9.69 × 10^4^ Da. FT-IR results showed that GTPS was composed of *β*-dominating configuration in pyranose form sugars. It has been well known that the natural plant polysaccharides are not only the major source of energy but also very important in our daily life as well. It has been found that the antioxidant activities of polysaccharides can be affected by a lot of factors such as structural features, molecular weight, configuration of glycosidic linkages, monosaccharide composition, and configuration, even the extraction and purification methods, which will certainly provide an opportunity to demonstrate the important biological roles of polysaccharides and develop a potential antioxidant food and medicine [26].

However, there are many other chemical compositions in *Yingshan Cloud Mist* Tea such as polyphenols, caffeine, theanine, and vitamins. Among them, polyphenols also have very high antioxidant capacity, mainly catechins and catechin derivatives. It has been reported that green tea catechins pose different physiological and pharmacological biological activities, including scavenging free radicals [27].

ROS are mainly generated by the body's normal oxygen utilization including cellular respiration and immune functions. Although most ROS can be removed by the body's endogenous antioxidant systems, excessive content of ROS can damage the components of cell such as protein, lipids, and DNA [28]. Scavenging excess of ROS depends on an efficient antioxidant system composed of nonenzymatic and enzymatic antioxidants [29]. Likewise, in poultry, oxidative stress is related to a lot of metabolic disorders that are observed under many stressful conditions, such as heat stress and diseases, and has been known as one of the major elements negatively affecting the performance of chickens in poultry industry [30]. In recent years, the antioxidant bioactivities of natural polysaccharides had received a wide attention in many regions. Because of the high toxicity and danger of synthetic antioxidants, most of the natural polysaccharides might be used as ideal antioxidant agents [31].

In this study, GTPS is one of the main active components in *Yingshan Cloud Mist* Tea and may be a good replacement of synthetic antioxidants. As we all know, human has a very complex antioxidant protection system to protect bodies from the damage of ROS, including the use of natural plant antioxidant. This system includes various components, endogenous or exogenous, which is capable of suppressing free radicals. These components are composed of antioxidant enzymes, metal binding proteins, diet rich in antioxidants like ascorbic acid, vitamin E, carotenoids, polyphenols, and other low molecular weight compounds [32]. However, some synthetic antioxidants were restricted in foods because of their carcinogenicity and toxicity.

As can be seen from our research, the antioxidant activities *in vitro* of GTPS were investigated including DPPH radical, superoxide anion, hydroxyl scavenging activity, and ferric-reducing power. The results showed that the antioxidant activities of GTPS and ascorbic acid increased in a dose-dependent manner and GTPS had a relatively high antioxidant capacity. One possible antioxidant mechanism might be because of the supply of hydrogen by tea polysaccharides, which combined with radicals and formed a stable radical to end the radical chain reaction. The other might be that tea polysaccharides could combine with the radical ions that were necessary for radical chain reaction, and then, the reaction was ended [33]. Some previous literatures showed that polysaccharides with antioxidant activity were associated with average molecular weights mainly distributed between 10 and 1000 kDa and composed of glucose, mannose, and galactose [34]. Wang et al. studied the antioxidant activity and *α*-glucosidase inhibitory effect of polysaccharides from three Oolong teas; the results indicated that a higher content of protein and uronic acid could result in higher antioxidant activities [35]. The antioxidant activities of polysaccharides were greatly affected by monosaccharide compositions [36]. Glucose and galactose took up a high proportion in GTPS, and the high proportion was very vital for the antioxidant activities [37].

*In vitro* experiment results revealed that GTPS had a considerably high antioxidant capacity, but there was a limitation that these data were obtained from *in vitro* assays. To further explore the application of GTPS, the study *in vivo* was needed. Next, the antioxidant test *in vivo* was conducted. GSH-Px and SOD were widely consisted in the antioxidant enzyme system in the animal bodies [38]. These protein behaviors could decrease the production of active oxygen radicals and inhibit lipid peroxidation and intermediate products of metabolism from causing damage. Total antioxidant capacity (T-AOC) was a main index to decide the total antioxidant level of the enzymatic and nonenzymatic system. T-AOC can eliminate free radicals and reactive oxygen species and further inhibit lipid peroxidation, reduce peroxidation materials, and remove metal ions from the catalytic reaction [39]. Malondialdehyde (MDA) is a main animal body product of lipid peroxidation. MDA can reflect the degree of oxidative damage and has cytotoxicity [40]. Serum has different kinds of antioxidant enzymes including SOD, GSH-Px, and CAT and can directly reflect the degree of antioxidant status in chickens when exposed to oxidative stress. Akhavan-Salamat and Ghasemi studied the effect of different sources and contents of zinc on antioxidant status of chickens through testing the serum antioxidant enzyme activities and MDA concentration [41]. Also, Li et al. investigated the effects of intermittent cold stimulation on antioxidant capacity in chickens by serum [42]. In the present study, the results indicated that GTPS could improve the antioxidant status of chickens. The basal diet supplemented with GTPS enhanced the capacity of antioxidant enzymes (GSH-Px, SOD) and the nonenzymatic system (T-AOC) and successfully inhibited lipid peroxidation as observed in the decrease of MDA content. Compared with the previous studies, the results obtained in this experiment were in great agreement with what were reported for natural polysaccharides in some literatures. For example, polysaccharide krestin (PSK) from *Coriolus versicolor* was reported to improve the oxidative damage by enhancing SOD and GSH-Px enzyme activities [43]. Choi et al. reported that natural polysaccharides could cause the reduction in the MDA level and the increase of SOD, GSH-Px, and T-AOC levels [44]. Li et al. investigated the antioxidant activities *in vivo* of water-soluble polysaccharides from Ostrea rivularis (ORPs), and the results indicated that ORPs significantly raised the level of T-AOC, reduced the formation of MDA, and enhanced the activities of SOD and GSH-Px [45].

Accordingly, GTPS could be used as a kind of natural antioxidant, and it may provide a reference for reducing the use of synthetic antioxidants. In addition, GTPS had the potential effect in promoting body weight gain in chickens, and it could be used as a kind of natural growth promoter as well.

## 5. Conclusion

Our present study showed that the molecular weight of GTPS was 9.69 × 10^4^ Da and composed of rhamnose, arabinose, xylose, mannose, glucose, and galactose with the molar ratio of 11.4 : 26.1 : 1.9 : 3 : 30.7 : 26.8. In addition, GTPS was capable of scavenging including DPPH radicals, superoxide anion, and hydroxyl radicals in a dose-dependent manner and enhanced the activity of ferric-reducing power. Then, diet with GTPS can increase the levels of GSH-Px, SOD, and T-AOC and decrease the content of lipid peroxidation product MDA in chickens. In addition, GTPS could enhance the chicken body weight. Taken together, these results demonstrated that GTPS could be used as a kind of natural antioxidant and be further developed in feed industry.

## Figures and Tables

**Table 1:** HPGPC results: molar weight of GTPS.

	Mp (Da)	Mn (Da)	Mv (Da)	Mw (Da)	Mz (Da)	Mw/Mn	Mz/Mw
GTPS	6.72 × 10^4^	3.63 × 10^4^	8.46 × 10^4^	9.69 × 10^4^	2.16 × 10^5^	2.67	2.23

Mp = peak average molecular weight. Mn = number-average molecular weight. Mv = viscosity average molecular weight. Mw = weight-average molecular weight. Mz = Z-average molecular weight.

**Table 2:** Effects of GTPS on growth performance in chickens.

Item	Groups			
Body weight (g)	NC	LDG	MDG	HDG
Body weight after prefeeding period	187.7 ± 5.57^a^	191.9 ± 4.03^a^	188.1 ± 4.24^a^	191.3 ± 4.40^a^
28 d	680.0 ± 30.00^d^	728.0 ± 37.09^c^	850.0 ± 28.28^b^	910.0 ± 30.00^a^
56 d	1092.0 ± 18.37^d^	1246.0 ± 64.65^c^	1486.0 ± 58.99^b^	1626.0 ± 97.11^a^
Average daily gain (g/d per chicken)				
1-28 d	17.58 ± 1.23^c^	19.15 ± 2.12^c^	23.64 ± 1.11^b^	25.67 ± 1.79^a^
28-56 d	14.71 ± 2.44^d^	18.50 ± 1.67^c^	22.71 ± 3.12^b^	25.57 ± 1.79^a^
1-56 d	16.14 ± 1.45^d^	18.82 ± 2.48^c^	23.17 ± 3.1^b^	25.61 ± 2.79^a^
Average daily feed intake (g/d per chicken)				
1-28 d	33.45 ± 2.12^d^	36.20 ± 1.56^c^	45.25 ± 2.47^b^	50.57 ± 3.22^a^
28-56 d	22.05 ± 3.71^d^	27.11 ± 2.65^c^	32.78 ± 2.26^b^	36.41 ± 3.16^a^
1-56 d	27.56 ± 1.98^d^	32.32 ± 2.67^c^	40.32 ± 2.61^b^	45.84 ± 1.34^a^
Feed conversion ratio (g/g)				
1-28 d	1.90 ± 0.06	1.89 ± 0.009	1.91 ± 0.01	1.97 ± 0.08
28-56 d	1.49 ± 0.005	1.47 ± 0.06	1.44 ± 0.008	1.42 ± 0.03
1-56 d	1.71 ± 0.008	1.72 ± 0.02	1.74 ± 0.01	1.78 ± 0.003

Within the same lines, values with different letters differed significantly (*P* < 0.05). The values were expressed as the mean ± SD (*n* = 15). NC: normal control group (basal diet). LDG: low-dose group (basal diet supplemented with 200 mg/kg GTPS). MDG: middle-dose group (basal diet supplemented with 400 mg/kg GTPS). HDG: high-dose group (basal diet supplemented with 800 mg/kg GTPS).

## Data Availability

The data used to support the findings of this study are available from the corresponding authors upon request.
